# Differences and similarities between cancer and somatic stem cells: therapeutic implications

**DOI:** 10.1186/s13287-020-02018-6

**Published:** 2020-11-18

**Authors:** Fiorella Rossi, Hunter Noren, Richard Jove, Vladimir Beljanski, Karl-Henrik Grinnemo

**Affiliations:** 1 NSU Cell Therapy Institute, Nova Southeastern University, 3301 College Ave, 3200 South University Drive, Fort Lauderdale, FL 33328 USA; 2 Department of Molecular Medicine and Surgery, Karolinska Institutet, Stockholm, Sweden; 3 Department of Surgical Sciences, Division of Cardiothoracic Surgery and Anaesthesiology, Uppsala University, Akademiska University Hospital, Akademiska sjukhuset, ingång 50, 4 tr, 751 85 Uppsala, Sweden

**Keywords:** Cancer stem cells, Somatic stem cells, Immunoregulation, Targeted therapy, Signaling pathways, Clinical trials, Cell surface markers, Cellular differentiation, Self-renewal

## Abstract

Over the last decades, the cancer survival rate has increased due to personalized therapies, the discovery of targeted therapeutics and novel biological agents, and the application of palliative treatments. Despite these advances, tumor resistance to chemotherapy and radiation and rapid progression to metastatic disease are still seen in many patients. Evidence has shown that cancer stem cells (CSCs), a sub-population of cells that share many common characteristics with somatic stem cells (SSCs), contribute to this therapeutic failure. The most critical properties of CSCs are their self-renewal ability and their capacity for differentiation into heterogeneous populations of cancer cells. Although CSCs only constitute a low percentage of the total tumor mass, these cells can regrow the tumor mass on their own. Initially identified in leukemia, CSCs have subsequently been found in cancers of the breast, the colon, the pancreas, and the brain. Common genetic and phenotypic features found in both SSCs and CSCs, including upregulated signaling pathways such as Notch, Wnt, Hedgehog, and TGF-β. These pathways play fundamental roles in the development as well as in the control of cell survival and cell fate and are relevant to therapeutic targeting of CSCs. The differences in the expression of membrane proteins and exosome-delivered microRNAs between SSCs and CSCs are also important to specifically target the stem cells of the cancer. Further research efforts should be directed toward elucidation of the fundamental differences between SSCs and CSCs to improve existing therapies and generate new clinically relevant cancer treatments.

One of the major problems in the failure of cancer treatments is the presence of cancer stem cells (CSCs)—they are considered to be responsible for drug therapy resistance and thought to be involved in cancer initiation and metastasis. However, a successful therapy should have minimal side effects on normal somatic stem cells (SSCs). Understanding the differences in origin, mechanism of self-renewal, and signaling pathways of CSCs and SSCs will provide a better approach to target these specific populations in order to protect healthy cells and minimize side effects.

## The origin of cancer cells

Cancer is one of the most pervasive causes of morbidity and mortality in the Western world; it is the second leading cause of death after cardiovascular disease, and one of the most pressing current problems in public health [1]. Cancer is described as a proliferative, invasive, and metastatic disease that is caused by an accumulation of genetic abnormalities that randomly produce a malignant cell [2]. Such abnormalities can be induced by chemical carcinogens, chronic inflammation, exposure to radiation, or by genetic predisposition [3]. Cancer originates when normal cells accumulate DNA mutations over time and lose the ability to grow and proliferate in a regulated manner, leading to unrestricted cell proliferation. Phenotypically and functionally, cancer cells are abnormal and unstable and show inter- and intra-tumor heterogeneity. Inter-tumor heterogeneity is manifested as the difference in tumor composition between different individuals for the same cancer type; while intra-tumor heterogeneity is described as differences between tumor cells inside of the same tumor [4]. Cancer cells can arise in almost any tissue but they are most commonly found in the breast, ovary, prostate, liver, stomach, pancreas, lung, brain, and bone marrow. Because of differences in genetic composition and oncogenic signaling, tumors in different tissues exhibit different behaviors. Pancreatic tumors, for example, have a tendency to be highly aggressive, while prostate tumors are more confined and easier to treat [5].

**Fig. 1:**
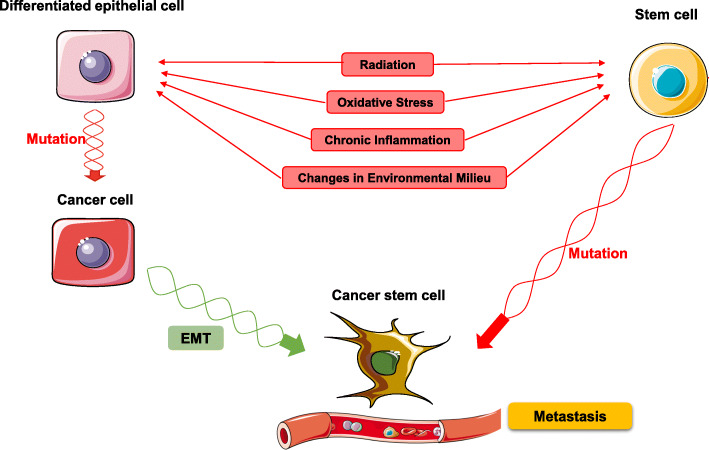
Role of environmental milieu and chronic inflammation in CSC maintenance. When epithelial cells are exposed to stressors such as radiation, oxidative stress, chronic inflammation, or changes in its environmental milieu, mutations in DNA can occur. While stem cells can mutate directly into CSCs, epithelial cells require a two-step process: first, the initial stress causes it to mutate into a cancer cell; then, it can undergo epithelial-mesenchymal transition (EMT) to become a cancer stem cell capable of metastasis

Cancer develops by the accumulation of mutations in genes leading to the deregulation of signaling pathways that initiate the acquisition of self-sufficient growth signals leading to insensitivity to anti-growth signals, evasion of apoptosis, unlimited replicative potential, sustained angiogenesis, and capacity to invade surrounding tissues [6]. The metastatic colonization is mainly accomplished by a sub-population of cancer cells that enter the blood stream allowing them to reach distant sites [7]. Clinically, cancer is classified according to a cancer-specific staging system, which together with the cancer type and grade are important parameters for successful treatment. The staging system is based on the size of the tumor, the extent of the lymph node spread, and the presence or absence of metastases [8]. Several genes have been shown to play a role in the initiation and progression of cancer. On a cellular level, cancer progression can be divided in different stages: tumor initiation, tumor progression, angiogenesis, and metastasis [9]. When these heterogeneous tumors are put under selection pressure by chemotherapy, a specific sub-population of resistant cells can selectively take over, allowing this sub-class to dominate the tumor [10]. Recently, it was identified that a small part of that heterogeneous population is composed of stem-like progenitor cells or CSCs that could originate from normal or stem cell mutations initiated by changes in the environment, by chronic inflammation, or by epithelial-to-mesenchymal transformation (EMT, discussed later) (Fig. 1) [11, 12]. Mutations in stem cell can occur by gradual accumulation of aging-induced genetic changes during lifetime [13]. Aging leads to a decrease of genome integrity resulting in increased cancer risk [14]. It was shown by Weinberg group that at least three to four cell type-specific mutations are require for cellular transformation in vitro [15, 16]; these mutations also increases exponentially with age and aging is associated with increased clonal dominance of mutant stem and progenitor cells [14].

The strongest evidence for the CSC theory comes from studies in acute myelogenous leukemia (AML), when Bonnet and Dick performed serial transplantation experiments to show that only rare cells with high self-renewal capacity isolated from AML patients could initiate leukemia in murine models. Later the cell responsible for tumor initiation was identified by its phenotype as CD34^+^CD38^−^, a remarkably common phenotype of a normal hematopoietic stem cell (HSC) [17]. Additional studies have supported the idea of CSCs and currently this idea is becoming commonly accepted [18, 19]. While CSCs might represent a small fraction of the cells in the heterogeneous tumors, they likely play a fundamental role in cancer initiation, progression, and metastasis as well as in therapy failure [20]. CSCs are also called tumor-initiating cells since they possess several important properties of SSCs: self-renewal, unlimited proliferation potential, slow replication, resistance to drugs, and the capacity to differentiate, giving rise to daughter cells, which make up the bulk of the tumor. While CSCs can initiate tumor formation, their daughter cells are incapable of forming new tumors by metastasis [21]. There is some controversy regarding the origin of CSCs: while in most instances they may originate from normal stem cells, there are some CSCs that can arise from somatic cells. Since their origin is not clearly understood, the term tumor-initiating cells have been used to define these cells. In general, CSCs are considered the seed of the tumor mass which also promotes its growth [22].

Intriguingly, Scadden showed that somatic cells, which give rise to cancer cells, do not need to have stem cell properties but that a stem-like phenotype could be acquired during the process of tumorigenesis, which is further an indication of the tumor cells ability to adapt to environmental factors [23]. In this review, we will focus on the most important similarities and differences between SSCs and CSCs to examine potential avenues to target CSCs with minimal impact on SSCs.

## The biological function of somatic stem cells

SSCs can undergo extensive cell division while retaining the ability to give rise to stem cells and cells that can differentiate into specialized cells [24]. Among the several characteristics of a SSC, the most important are self-renewal and multipotent differentiation capacity. Self-renewal is defined as a special cell division that enables stem cells to produce another stem cell with the same replication potential [25]. Self-renewal is achieved in response to systemic or local signals that induce cell proliferation while maintaining tissue-specific properties [25, 26]. Another important function of SSCs involves cell differentiation of daughter cells into tissue-specific specialized cells, i.e., mature cells of a specific tissue [27]. SSCs repair damaged tissues and maintain normal tissue homeostasis by replenishing many cells throughout an organism’s life [28, 29]. In addition, SSCs can be constantly under division, such as those found in the gut and bone marrow or can remain quiescent (non-dividing) for prolonged periods of time until activated [30]. Once activated by signals originating in their microenvironment, SSCs can contribute to tissue repair by differentiating into specialized cells by the action of paracrine and autocrine signals [31].

Stem cells can be classified according to their exclusive differentiation potential: totipotent (they can generate all cell types in the body including extra-embryonic tissue or placenta), pluripotent (they can generate all body cells including germ cells), multipotent (they can be further specialized in the tissue), and unipotent (they only generate a single cell type) [32]. Depending on the stage of embryonic development that the embryonic stem cells (ESCs) are derived, these can be totipotent or pluripotent [33], while adult or SSCs are shown to be multipotent [34].

ESCs and SSCs can be distinguished according to specific intracellular and surface markers. There are some transcription factors that are commonly expressed in ESCs, the so-called core nuclear factors, like Oct-3/4, Sox2, KLF4, and Nanog [35]. A wide range of cell surface markers characterizing ESCs have been reported, among the most common are the cluster of differentiation (CD) antigens surface proteins. CD antigens associated with pluripotency are CD9, CD24, and CD133. Additionally, ESCs express CD90, CD117 [36]. ESCs also express specific integrins that play a role in cell adhesion, signaling and cell migration. The most important are CD324 (E-cadherin) and CD29 (β1 integrin). Other proteins like TRA-1-60 and TRA-1-81, Frizzled5, and Cripto-1 are also characteristic of ESCs [36].

SSCs are thought to be tissue-specific, which means that they give rise to progeny that correspond to their tissue of origin. SSCs can be found in adult tissues like the intestine, skin, muscle, blood, and nervous system [37]. The two clinically utilized SSCs are HSCs and mesenchymal stromal cells (MSCs). HSCs are derived from the mesoderm and can be found in bone marrow and umbilical cord blood, and they give rise to blood cells during hematopoiesis and can be used in hematopoietic cell transplantation [38, 39]. On the other hand, MSCs are progenitor cells that give rise to cells representing different mesenchymal lineages and can be found in virtually any tissue but especially adipose, bone marrow, umbilical cord, and possibly in the human testis [40]. The minimal criteria for MSCs are that they should be plastic-adherent in standard culture conditions, expression of CD73 (SH3), CD90, and CD150 (SH2) and lack of expression of CD11b, CD14, CD19, CD34, CD45, and HLA-DR molecules and be able to differentiate into chondrocytes, osteoblasts and adipocytes in vitro [41]. MSCs are currently being explored in clinics in treatments of various conditions, including graft versus host disease, cardiovascular diseases, and several autoimmune diseases [42].

## Key properties of CSCs

CSCs share the same cellular and molecular mechanisms that regulate SSCs; however, CSCs lack the necessary control system to prevent uncontrolled proliferation [43]. While the specific origin of CSCs is still under debate, evidence suggests that they originate from stem cells that failed to control proliferation under abnormal circumstances [44, 45]. Other proposed origins suggest that CSCs could arise from cell-cell fusion between cancer cells and adult stem cells, gene transfer between somatic and cancer cells, or mutations in stem cells [46]. In addition, transformation could occur during the process of tissue regeneration in response to inflammation, infection, toxin exposure, and/or metabolic processes which could cause mutations [47].

CSCs have been identified in several solid tumors based on the expression of certain CSC surface markers. Until now, CSCs have been identified by surface markers that are common between different cancer types: CD24, CD29, CD44 CD90, CD133, aldehyde dehydrogenase 1 (ALDH1), and epithelial-specific antigen (ESA) [48–50]. Depending on the type of tissue from which they originate, they can express a variety of markers for each type of CSCs. Most importantly, the expression of these markers can be used for specific therapeutic targeting of CSCs [51] (further discussed in later sections).

Evidence from a xenotransplantation experiment of human brain tumors into non-obese diabetic severe combined immunodeficiency (NOD/SCID) mouse brains did demonstrate the presence of CSCs in the tumor fraction responsible for tumor regeneration [52]. Other studies also report that certain types of cancers, such as hepatocellular carcinoma (HCC), can be derived from SSCs [53]. HSCs originate from self-renewing progenitors in the bone marrow where they mostly reside; however, they also can be found in peripheral blood. Clinical and genetic evidence suggest that certain types of leukemia are driven by genetic mutation in HSCs. These mutations give rise to leukemia stem cells, also called CSCs of the blood [54, 55]. Additionally, in a NOD/SCID murine model of leukemia, phenotypic differences between leukemia stem cells and HSCs were shown in separate studies [56, 57]. Recent efforts uncovered surface markers that are exclusive for AML stem cells such as CD123, TIM3, CD47, CD96, CLL-1, and IL-1 receptor accessory protein. On the other hand, AML stem cells rarely express CD90 and CD117 on their surface [58] and several studies have reported that CD123 was predominantly expressed in a subset of AML stem cells characterized by CD34+/CD38− phenotype [56]. These basic differences in the phenotype of CSCs could be essential to providing new avenues for the development of targeted cancer therapeutics.

CSCs are commonly confused with cancer-initiating cells because of their stem cell-like properties, where both are characterized by elevated expression of the stem cell surface marker CD133 [59]. When cancer-initiating cells receive the first cancer-causing mutation, they are hypothesized to be different from SSCs; however, they show several similarities such as low proliferative rates, high self-renewal, and resistance to chemotherapy and radiation [60]. Currently, it is not clear whether CSCs originate from cancer-initiating cells or if both cell types have the same origin. However, both cells support tumor initiation and propagation.

### Environmental milieu and chronic inflammation support CSCs

Cell microenvironment is fundamental for cell growth, fate, and interaction with other cells in response to a specific stimulus. Recent studies have confirmed that the microenvironment can support generation and growth of solid tumors [61], and it is possible that alterations in paracrine signals from niche cells could initiate or enhance tumor formation from SSCs. These signals function as a stimulus to induce activation, differentiation, proliferation, and/or cell death [62]. Moreover, these environmental stimuli are a part of a greater structure called “stem cell niche”. This niche refers to a specific microenvironment inside a discrete anatomic location where SSCs are found in an undifferentiated and self-renewable state [63]. These niches have been observed in different mammalian epithelial tissues, in the gastro intestinal tract, and in the neural and hematopoietic system where they regulate stem cell fate by directing cell-cell interaction and secretion factors [64]. Soluble factors secreted from primary tumors can stimulate the recruitment of cells to the niche. Growth factors such as VEGF, TGF-β, and TNF-α have been identified as the major factors secreted from primary tumors which promote angiogenesis [65, 66].

Soluble factors released in the microenvironment strongly influence the growth of a primary tumor, where its growth is further enhanced by changes in the niche environment [64]. CSCs reside in these niches which are responsible for maintaining the self-renewal capacity and undifferentiated state of CSCs [67]. These niches maintain the main properties of CSCs by preserving their phenotypic plasticity, protecting the cells against the immune system and low nutrient availability, resisting oxidative stress, detoxifying drugs via ATP-binding cassette transporters, and promoting metastasis [68]. These niches also favor the recruitment of more cells to induce inflammation by secreting factors (cytokines and chemokines). The secreted factors facilitate the formation of secondary and tertiary tumors [69], where CSCs disseminate through the stroma into the bloodstream and induce metastasis [70].

Another important environmental factor driving tumor progression is chronic inflammation. This condition is hypothesized to be one of the principal factors in CSCs expansion and tumor dissemination [71]. This inflammatory response can be initiated by the activation of toll-like receptors (TLRs), stimulated by pathogen-associated-molecular patterns (PAMPs) of carcinogenic microbes or by products released from cancer cells. Consequently, nuclear factor kappa B (NF-κB) is activated inducing an inflammatory response that could increase self-renewal activity in cancer cells [72]. It was shown that in CD44^+^/MyD88^+^ epithelial ovarian CSCs, the TLR2/MyD88/NF-κB signaling can support its growth and self-renewal by the upregulation of stemness-associated genes [73]. Another TLR that seems to promote the expression of stemness-associated genes is TRL3. For example, Jia et al. showed that poly(I:C) enhances stemness in cancer cells by the mutual activation of β-catenin and NF-κB signaling pathway. They demonstrated both in vitro and in vivo that breast CSCs, characterized by expression of CD44high/CD24−/low markers, possess stem cell-like properties and tumor-initiating capacity. They also established that TLR3 activation in breast cancer cells contributes to an enrichment of a subset of cells with the CSC phenotype [74].

Further evidence that supports the role of inflammation in cancer progression stems from observations that malignant tumors often develop at sites of chronic inflammation and tissue injury [75]. Chronic viral hepatitis [76], general gastric inflammation [77], gastritis caused by *Helicobacter pylori* [78], inflammatory bowel disease, and several other chronic inflammation conditions are also shown to increase the risk of cancer development [79], and the induction of CSCs [80]. Tumor stroma also contains activated fibroblasts, inflammatory cells, and nascent blood capillaries. The formation of such microenvironments facilitates induction of an inflammatory response that causes cell migration and epithelial cell proliferation. This results in tissue repair that can occasionally turn into uncontrolled cell proliferation and dissemination [81, 82]. O’Brien et al. showed that the ability of cancer cells to function as CSCs depends on how they respond to the self-renewal signals released in the environmental milieu [83]. It was suggested that changes in the environmental milieu can lead to reprogramming of SSCs turning them into cancer stem cells after prolonged inflammation, infection, exposure to toxins, or autoimmune diseases [84]. Some reports have shown that tumor environmental milieu provides the stimuli necessary for the transformation of SSCs by secreting TGF-β [85]. This cytokine will enhance the transition from SSCs to CSCs by inducing zinc finger E-box-binding homeobox1 (ZEB1) transcription factor expression. ZEB1 contributes to cancer dissemination and the activation of epithelial-mesenchymal transition (EMT), a process that has been linked to cancer metastasis. Additional evidence suggests that ZEB1 is responsible for the maintenance of CSC-like phenotypes [86, 87]. Another example of inflammatory conditions affecting CSC development is hepatocellular progenitor cancer cells (HcPCs), which have been observed following chronic inflammation in the liver. HcPCs show a similar transcriptomic profile to the SSCs in the liver but these cells do not generate tumors. However, under chronic inflammation, interleukin-6 (IL-6) secretion stimulates HcPC growth in vivo and facilitates tumor progression [88]. The role that chronic inflammation plays in the induction of different types of CSCs is still under investigation, but cytokines secreted by tumor-associated immune cells seem to activate the necessary pathways required by cancer cells to become cancer stem cell like.

## Immunoregulatory properties of SSCs and CSCs

One important characteristic of SSCs is their ability to regulate the immune response during inflammatory conditions. The immune system is designed to recognize foreign antigens expressed on antigen presenting cells (APCs). This recognition leads to the activation of naïve T cells [89], which involves the specific recognition of a T cell receptor with a peptide bound to a major histocompatibility complex (MHC) class II [89]. Two signals are fundamental to ensure the appropriate activation: one is the interaction of MHC class II loaded peptide and T cell receptor (TCR), and the other signals are provided by co-stimulatory molecules CD80, CD86, CD40; adhesion molecules such as lymphocyte function-associated antigen 1 and intercellular adhesion molecule 1. There are also negative co-stimulatory molecules that are responsible for T cell suppression [90]. These suppressive co-stimulatory molecules or negative regulators of T cell immune function molecules are programmed death ligand-1 and cytotoxic T-lymphocyte associated antigen-4.

MSCs are weakly immunogenic and possess immunomodulatory properties toward natural killer cells, dendritic cells, neutrophils, B cells, and T cells [91], where the effect on T cells are the most studied. MSCs exert their immunoregulatory effects by inhibiting activated T cell proliferation as well as stimulating regulatory T cell (Treg) proliferation [91]. For example, MSCs decrease the T helper 1 (Th1) response [92, 93], but they can also induce a shift from Th2 to Th1 response under certain inflammatory conditions [94, 95], suggesting that MSCs can switch their phenotype to protect the body from disease in different situations. Mechanistic aspects of MSC-mediated suppression of T cells reveal three complementary mechanisms: (1) cell-cell contact interaction between MSCs and T cells, (2) secretion of soluble mediators, and (3) generation of Tregs (reviewed in [96]). Recognizing these properties opens a field for treatments that tackle immune response modulation. Pre-clinical animal models of large-organ transplant rejection, autoimmune diseases, and chronic inflammatory diseases showed that allogeneic MSCs induced a marked suppression of the host immune response [91]. Additionally, some clinical trials that used MSCs in patients with severe graft-vs-host disease (GVHD) showed that the administration of MSCs resulted in an improvement in the clinical response [97]. It was also demonstrated that MSCs modulate GVHD by CD4^+^ and CD8^+^ T cell proliferation suppression, B cell suppression, Tregs induction, Th17 suppression, and the release of soluble immunomodulatory factors [98]. Similar positive effects of bone marrow MSCs have also been demonstrated in patients with acute respiratory distress syndrome (ARDS) [99, 100]. Besides these conditions, MSCs are widely used in clinical trials of spinal cord injuries, Sjögren’s syndrome, rheumatoid artritis, lupus erytromatosis, amyotrophic lateral sclerosis, multiple sclerosis, and neuropathies.

As previously discussed, the immunoregulatory properties of MSCs are well studied; however, little is known about the potential immunological properties of CSCs. It is well known that the immune system plays an important role in attacking tumor cells [67] by detecting traits of malignant transformation by immune cells. Conversely, tumor cells can sometimes escape immune recognition, especially when progenitor cancer cells have already been established [101]. It has been shown that CSCs are more tumorigenic than regular cancer cells found in the solid tumor [102] and CSCs might evade the host immune response due to their phenotypic and functional properties, allowing them to endure and spread throughout the body. Tumor cells can evade the host immune response by different mechanisms, among them are the production of immunosuppressive factors, low expression of tumor antigens, lack of expression of human leukocyte antigens (HLAs), and co-stimulatory molecules [103]. Immunosuppressive factors are produced in the tumor microenvironment where other immune cells also participate by releasing soluble factors that further immunosuppress the environment [104]. The action of tumor-associated macrophages (TAM), an activated M2 polarized population, suppresses the inflammatory response and promotes tumor angiogenesis [105]. It was shown that TAM co-localized with CD133^+^ glioma cells increased the invasive capability of these cells by secreting TGF-β1 [104]. Little is known about CSCs immune-regulatory properties but certainly they represent an important factor in the failure of cancer treatment. It was suggested that CSCs can escape natural killer (NK) cells recognition by entering into a latency stage and downregulating ULBP ligands that binds to and activates NK cells [106]. Additionally, CSC can evade cytotoxic T cells by downregulating MHC-I and upregulating the expression of PD-L1, resulting in host immunosuppression [107].

A study compared the immunological properties of CSCs-like cells (CD44^+^) with tumor cells (CD44^−^) and demonstrated that CD44^+^ cancer stem-like cells have an immunosuppressive phenotype. This effect was probed by the inhibition of activated T cell proliferation, the induction of Treg polarization, and the suppression of Th1, which leads to the dysfunction of effector T cell and cytotoxic T cells [103]. Another study showed that CSCs isolated from therapy-resistant tumors have pro-inflammatory tumorigenic properties; such CSCs can induce macrophage colony-stimulating factor production by activating interferon regulatory factor-5 (IRF-5) pathway [108]. This study also found that they could induce several pro-inflammatory cytokines and chemokines such as TNF-α, IL-6, and IL-8, creating a tumorigenic microenvironment [108]. Recent studies also suggest that, in addition to secreted cytokines, other secreted factors also contribute to downregulation of the immune response. For example, extracellular vesicle-mediated communication between tumor cells and immune cells may result in downregulation of the immune response to tumor cells [109]. Bi et al. accentuated the role of IRF-5 in regulating tumor infiltration and found that the loss of IRF-5 expression in human ductal carcinoma correlates with disease stage and contributes to metastasis [110]. Therefore, it appears that MSCs and CSCs share the same immune-regulatory properties. The difference is that MSCs use these properties in regeneration of damaged tissues and in immunomodulation, while CSCs use this property to endure in the tumor and evade the immune response. These characteristics also suggest that immune-suppression pathways could potentially be targeted for tumor clearance by immune response mediated mechanisms.

## Self-renewal activity and signaling pathways in CSCs

Similar signaling pathways regulate the self-renewal activity in ESCs, SSCs, and CSCs. ESCs are pluripotent and can differentiate into any specialized cell in the body, while SSCs have more limited differentiation capacities [111]. ESCs can be maintained in culture for long periods of time without losing their undifferentiated state [112]. On the contrary, SSCs cultured in vitro differentiate rapidly, indicating that environmental and internal signals are fundamental for SSCs differentiation process and self-renewal [113, 114]. It is suggested that internal signals directed by genetic and epigenetic processes, and external signals composed of secreted cytokines and growth factors can change the fate of SSCs [86]. The self-renewal activity of SSCs is highly regulated by different signaling pathways but this tight regulation is lost in CSCs. It has been shown that specific pathways such as Wnt/ β-catenin, Jack/Stat, TGF-β, Notch, and Sonic Hedgehog are deregulated in CSCs [115]. Instead, CSCs have the ability to use these self-renewal pathways to drive tumor dissemination.

### Role of Wnt/β-catenin pathway in CSCs

The Wnt signaling pathway belongs to a family of secreted glycoproteins that have several functions, including regulating proliferation, differentiation, and patterning throughout embryonic development [116]. Other components of the Wnt pathway include molecules of the Wnt secretory machinery, Wnt co-receptors, new components of the β-catenin degradation machinery, and nuclear co-factors [117, 118]. Mutations in Wnt genes or defects in their signaling pathway cause specific developmental defects during embryonic development leading to certain human diseases and cancer during adulthood [119–122]. Abnormal activation of the Wnt/β-catenin pathway is one of the most frequent abnormalities present during tumorigenesis. Abnormal expression of Wnt ligand proteins has been observed in different types of tumors in osteosarcoma [123], hematological malignancies [124], breast cancer [125], and non-small cell lung cancer [126]. Malanchi et al. showed that β-catenin signaling is essential in maintaining CSC phenotype and its inhibition results in the loss of CSCs and total tumor regression indicating increased activation of β-catenin pathway in human squamous cell carcinoma [127]. Moreover, Wnt signaling has been shown to be deregulated in leukemic stem cells compared to somatic hematopoietic stem cells. These results were obtained from a genome-wide expression analysis that compared the expression profile of highly enriched normal human HSCs and leukemic stem cells from patients with AML [128]. In AML, deregulated activation of the Wnt/β-catenin pathway induces cell proliferation by turning on genes encoding oncoproteins and cell-cycle regulators [129]. Furthermore, Wnt5a pathways seem to be involved in the regulation of CSCs, as reviewed by Zhou et al. which showed that there is a complex cross-talk between Wnt5a and specific receptors that are expressed in ESCs and that may be expressed or activated in CSCs but not in SSCs [130].

### STAT signaling deregulation is associated with CSCs induction

Signal transducers and activators of transcription (STATs) involve tyrosine phosphorylation by Janus family tyrosine kinases (JAKs) that further allow STAT protein dimerization and nuclear translocation and, finally, expression of target genes [131]. Gene encoding of the STAT family is localized on different chromosomes: in chromosome 2 (STAT1 and STAT4), in chromosome 12 (STAT2 and STAT6), and in chromosome 17 (STAT3 and STAT 5A/B) [132]. JAK-STAT signaling pathway regulates somatic cell differentiation, proliferation, immune response, and apoptosis [133]. We previously discussed the role of JAK-STAT3 signaling pathway in promoting cancer through inflammation, obesity, stem cells, and pre-metastatic niche, and how the regulation of this pathway in tumors constitute an important target for therapeutics in the treatment of cancer [134]. We also found that STAT3 activation mediates the loss of androgen receptor expression, which promotes a stem-like cell phenotype in prostate cancer [135]. STAT signaling pathway has also been shown to regulate both stem cell-renewal and tumorigenesis, and abnormal activation of the STAT pathway induces cell transformation and oncogenesis of many cancer types [136, 137]. This deregulation of STAT pathway can cause the inhibition of differentiation pathways and instead induces stem cell self-renewal [138]. Moreover, it has been shown that STAT signaling plays a role in different stem cell niches in SSCs and CSCs. For example, STAT5 was shown to be activated in HSCs by the stem cell factors c-Kit and thrombopoietin, both fundamental factors for HSCs self-renewal [139]. STAT5 has also been observed to be constantly expressed in different hematological and non-hematological malignancies [139]. Hernandez-Vargas et al. suggested that a sub-population of sorted CD44^+^/CD24^−^, considered to be breast CSCs, constitutive activate JAK-STAT signaling pathway. Their data also support the concept that the expression of cancer stem-like pathways and the establishment and self-renewal properties of cancer stem cells are coordinated by epigenetic mechanisms [140].

### TGF-β pathway is involved in CSCs development and tumor progression

The TGF-β pathway is another important pathway involved in embryonic development, in adult tissue homeostasis, and in the regulation of stemness of CSCs [141]. TGF-β signaling is initiated upon TGF-β ligand interaction with type II and type I transmembrane serine/threonine kinase receptors on the cell surface which induces oligomerization of the receptor kinases and phosphorylation of the cytoplasmic signaling molecules Smad2 and Smad3 for the TGF-β/activin pathway [142]. The activated Smad complexes are translocated into the nucleus together with other nuclear co-factors to regulate the transcription of target genes [142]. Interestingly, the loss of function of certain Smads is observed during tumorigenesis of pancreatic and colorectal cancers as well as other cancer types [142–144].. TGF-β is mostly known for its role as hepatic pro-fibrogenic cytokine predominantly produced by activated mesenchymal cells upon chronic liver damage, suggesting that it also participates in tissue repair and maintenance in SSCs. TGF-β induces Smad3-dependent nuclear accumulation of β-catenin in MSCs, and it was shown that TGF-β signaling regulates the differentiation fate of MSCs [141]. Together with CSCs, TGF-β participates in the initiation and development of various tumors, and in the acquisition of CSC-like properties [145].

### Notch and Sonic hedgehog signaling are important in CSCs development

Notch signaling pathway also plays an important role during embryogenesis and cancer development [146]. Notch signaling is initiated by the interaction between a Notch ligand and a Notch receptor expressed on the surface of neighboring cells [147]. In mammals, the Notch pathway is formed by five canonical type I transmembrane ligands, including Delta-like ligands (DLLs), DLL1, DLL3, and DLL4; two Jagged proteins, Jagged1 and Jagged2; and four Notch transmembrane receptors, Notch1–4. This interaction triggers a two-step proteolytic cleavage of the receptor, mediated by an ADAM/TACE (tumor necrosis factor alpha converting enzyme) metalloproteases. ADAM10 and ADAM17 interact with nuclear factors to regulate target gene expression that regulates cell differentiation [115, 146]. This pathway is mainly involved in cell to cell communication, tissue differentiation, and self-renewal of stem cells. Notch pathway is deregulated in different cancers, and it is believed that Notch regulates the formation of cancer stem cells and the initiation of epithelial-mesenchymal transition phenotype [148].

Sonic hedgehog or hedgehog (Hh) signaling pathway was initially discovered in the fruit fly as a regulator of body segmentation in 1980. Hh signaling pathway forms one of the networks of major regulators of cell differentiation, proliferation, and cell polarity [149]. This pathway is a fundamental part of embryonic development [150]. It was shown that mutations generated in the Hedgehog pathway resulted in defective axial patterning, including cyclocephaly or holopronsencephaly, a cephalic disorder in which the Prosencephalon fails to develop in two hemispheres during embryogenesis [150]. Finally, it was discovered that alterations in Hh signaling pathway were also associated with cancer development [18]. Hh signaling has been shown to be crucial for the maintenance and expansion of CSCs. In other words, deregulation of Hh signaling has been linked with development of CSC formation and EMT development. This is seen in different types of tumors, especially in gastrointestinal cancers, leukemia, medulloblastoma, lung, and pancreatic cancer [151, 152]. Hh, like the Wnt signaling pathway, is one of the major regulators of cell differentiation and proliferation [153]. It has been shown that inhibition of Hh signaling depresses self-renewal of pancreatic cancer stem cells and reverses chemoresistance. Specifically, they found that PANC-1 tumorsphere has properties of stemness and differentiation and is highly tumorigenic [154]. All these pathways, in addition to their role in ESCs, SSCs, and CSCs self-renewal, have an important function as modulators of epithelial-mesenchymal transition initiation in cancer cells.

## Role of EMT and MET in CSC establishment and progression

**Fig. 2:**
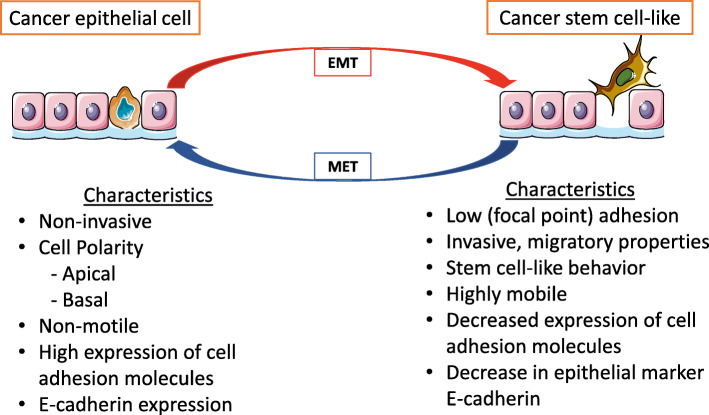
Role of epithelial-mesenchymal transition (EMT) in inducing cancer cell-like phenotype. Cancer epithelial cells can undergo EMT to induce a cancer stem cell-like phenotype that expresses characteristics of mesenchymal stem cells. They can also revert back, using a process called mesenchymal epithelial transition (MET) in which they reacquire epithelial cell characteristics. These processes contribute to cancer cells invasive capacities and new cancer initiation

EMT is a phenomenon observed during normal embryonic development and tissue repair and is characterized by epithelial cell losing their cell polarity, cell to cell adhesion, and gaining migratory properties and eventually transforming into MSCs [155]. While epithelial cells are connected to each other by tight junctions, gap junctions, and desmosomes [156], MSCs do not form tight junctions and have a migratory role. Other important characteristics of epithelial cells are that they have cell polarity, that they have an apical and basal orientation, and that their morphology is basically symmetrical [156]. EMT and its opposite process called mesenchymal-to-epithelial transition (MET) are important processes that occur during embryonic development [157, 158]. Besides its role during embryonic development, it is known that EMT occurs in other physiological process such as wound healing, and in the development of organ fibrosis [159]. Unfortunately, the EMT process has shown to be involved in cancer initiation and metastatic progression (Fig. 2) [160]. Tumor metastasis is a complex process where initial tumor cells disseminate from their primary site to a distant site where they form secondary tumors. It is believed that EMT contributes to cancer metastasis by facilitating local invasion, intravasation, transport, extravasation (by allowing cells to move to nearby blood vessels), and finally colonization [161]. During cancer, EMT and MET show a dynamic relationship in which cells transiently undergo MET and in the next step undergo EMT to restart the metastatic process [162]. A study by Yamamoto et al. showed that spatiotemporally coordinated mutual regulation between EMT and MET could occur during metastasis [162, 163].

It is believed that the EMT process is an important regulator of cancer cell plasticity and multipotency, supported by the connection of EMT-stem cells occurring in both normal epithelial cells and cancer cells [164]. This theory argues that cancer cells do not create a de novo stem cell program, but instead they adopt stem cell properties by inducing EMT. This confers mesenchymal traits to cancer cells turning them into cancer stem cell-like cells with properties of high motility, invasiveness, and self-renewal capable of colonizing other tissues [164].

### EMT induction and regulation by stem cell pathways

Epithelial to mesenchymal transition involves fundamental changes that involve several regulatory networks. EMT is triggered by the zinc finger protein SNAI1, Twist-related protein 1 (TWIST1), zinc finger E-box-binding homeobox 1 (ZEB1), teratocarcinoma-derived growth factor-1 also known as Cripto-1, TGF-β, Wnt/β-catenin, and Notch [165, 166]. Most of these pathways downregulate the expression of E-cadherin, resulting in the loss of E-cadherin-dependent intracellular epithelial junctional complexes [158]. Snail, a family of zinc finger transcription repressors, is considered a key inducer of EMT but also plays an important role in cell survival, immune regulation, and stem cell biology [167]. Snail together with ZEB1 represses E-cadherin and simultaneously represses other junction proteins transcription factors such as claudins and desmosomes [160]. Additionally, ZEB1 was shown to be an important factor regulating cell plasticity and the induction of metastasis. On the contrary, ZEB1 depletion suppresses stemness and colonization in tumor cells [168]. Zhou et al. demonstrated that Snail contributes to the maintenance of stem cell-like phenotype in human pancreatic cancer by using a murine xenograft model of Snail knockdown [169]. They also showed that Snail knockdown led to a reduced number of tumor-bearing mice and a reduced average size of tumors that highly expressed E-cadherin and low expression of Oct4, a common transcription factor in stem cells, indicating that Snail is required in the preservation of the stem cell-like phenotype of cancer cells in pancreatic cancer [169]. Additionally, it has been shown that Snail-induced EMT is involved in promoting cancer stem cell-like properties in head and neck cancers [170]. Another important transcription factor involved in cell lineage fate and differentiation is TWIST1 also known as class A basic helix-loop-helix protein 38 transcription factor [171]. TWIST1 seems to be an important factor that contributes in tumor initiation by conferring cancer cell with stem-like properties. TWIST1 is also a key player in the induction of EMT by promoting invadopodia-mediated extracellular matrix degradation [171].

Notch, TGF-β, and Wnt/β-catenin signaling are known to participate in stem cells self-renewal pathways and have been proven to be involved in the regulation of CSCs and the initiation of EMT. All these pathways seem to be interconnected, as in the case of Notch and TGF-β pathway. These two pathways aid in the EMT cross-talk with different transcription and growth factors such as Snail, Slug, and ZEB1 [172]. An in vitro study showed that the overexpression of Notch-1 increases the expression of Snail in immortalized endothelial cells and causes Snail to bind to the E-cadherin promoter region, resulting in E-cadherin gene repression [173]. Moreover, Notch1 increases TGF-β/Smad signaling by upregulating the expression of TGF-β and TGF-β type 1 receptor, supporting the role of TGF-β in the induction of EMT and survival of CSCs. The stimulation of TGF-β receptors leads to the expression of the mesenchymal genes, such as vimentin, in addition to inducing ZEB1 [174]. Wnt signaling pathway mediates the initiation EMT process by the maintenance of mesenchymal state and stem cell properties promoting the stabilization of cytoplasmic β-catenin. Taken together, there is a reciprocal regulatory loop between the different pathways that cooperatively regulate EMT and is also associated with self-renewal activity in cancer stem-like cells or CSCs.

### Regulation of EMT by microRNAs

MicroRNAs (miRNAs) are molecules consisting of approximately 21–25 nucleotides. They post-transcriptionally regulate gene expression to maintain biological homeostasis and regulate immune response [175–177]. Even though miRNAs seem to have an important role in self-renewal of SSCs, they also appear to play an important role in EMT regulation and CSCs initiation [178]. Some experiments using murine embryonic stem cells have shown that a knockout of DGCR8 and Dicer1, two major components of the miRNA silencing complex, causes a defect in stem cell differentiation [175–177]. According to Chakraborty et al., there are six major factors that are required for pluripotency maintenance, Nanog, Sox2, Oct4, KLF4, Lin28, and c-Myc [179, 180].. MiRNA-296, miRNA-470, miRNA-134, and miRNA181a were postulated to be potential targets for these factors and to inhibit stem cells self-renewal [181].

Besides its role in self-renewal, miRNAs also play an important role in different aspects of tumor progression, cancer cell invasion, metastasis, and EMT [182]. miRNA dysregulation has been shown in different types of cancers [183], such as breast, prostate, lung, and gastrointestinal cancers [184–186]. EMT is a complex process that is regulated by several signaling pathways in which miRNAs could potentially regulate directly by binding and suppressing EMT transcription factors, or indirectly by binding to an inhibitor of EMT. For example, miRNA-200 family, including miRNA-200b, miRNA200c, and miRNA-141, are some of the most important regulators of EMT [187]. It was shown that miRNA-200 targets ZEB1 and ZEB2 (E-cadherin repressor) and inhibits EMT progression [188]. The ectopic expression miR-200 caused the upregulation of E-cadherin in cancer cell lines and reduced their motility, while its inhibition reduced E-cadherin and vimentin expression, and induced EMT [187]. Other studies also showed that downregulation of miRNA-200 induced an increase in EMT in normal breast cells as well as in CSCs [148]. miR-21 downregulation in breast cancer cells caused a reversal of EMT and a decline in CSC numbers [148]. Additionally, other miRNAs that bind to transcription factors involved in EMT are miRNA-30, miRNA-34, and miRNA-203, blocking EMT induction. Taube et al. showed that epigenetic silencing of microRNA-203 is necessary for the induction of EMT and to obtain cancer stem cell properties. They also suggested that restoring miRNA-203 expression levels could inhibit metastasis [189]. On the other hand, there are other miRNAs that promote EMT. For example, miRNA-9 binds and suppresses E-cadherin, while the expression of miRNA-424 was showed to be linked to EMT induction in prostate cancer.

## Therapeutic strategies to target CSCs

### Targeting cell surface markers commonly expressed in CSCs

A potential breakthrough in targeting CSCs could be achieved through targeting the differences in surface expression markers between SSCs and CSCs. Even though CSCs from different tissues have been characterized according to their phenotypic differences, there is still no definitive marker that only targets a specific population of CSCs. Currently, the identification and isolation of CSCs continue to be a challenge for therapeutic development; however, certain markers have been shown to be highly expressed among several different CSCs. Targeting human surface expression markers with monoclonal antibodies has shown to be a clinically and commercially established therapy. In CSCs, a combination of several markers could be the best approach for specific targeting of CSCs; for example, in breast cancer, the most highly expressed CSC markers include CD133, CD44, and aldehyde dehydrogenase (ALDH) [190]. CD133 (prominin-1) is expressed on CSCs in multiple tumors, and high expression of this marker has been associated with poor cancer prognosis, making this molecule a good target candidate [191]. CD44, a glycosylated type-1 transmembrane glycoprotein, is involved in cell to cell interactions, cell proliferation and cell migration, among the most widely used CSC markers, it is associated with increased potential for tumor initiation and progression [192]. Jin et al. showed that targeting CD44 with neutralizing antibodies inhibited tumor proliferation by eradicating human AML stem cells [193]. Both CD133 and CD44 have been identified on CSCs in breast, brain, colon, lung, prostate, liver, and gastric cancers, which makes these markers a potential target for neutralizing antibodies, antibody-mediated cancer stem cell therapies, or by engineering exosomes expressing these markers for drug delivery.

ALDH is an enzyme that catalyzes the oxidation of aldehydes to carboxylic acids to protect cells from oxidative stress. ALDH has been shown to be important for the maintenance of normal HSCs, and it is also commonly used as a marker to differentiate CSCs from different cancers [194]. Specifically, ALDH-1A1 subtype is frequently expressed in tumor-initiating cells or CSCs in several malignancies. Visus et al. generated ALDH-1A1_88–96_ peptide-specific T cells for immune-targeting ALDH-1A1 expressing CSCs resulting in specific T cell targeting that controlled tumor growth and metastasis [195].

As shown in Table 1, several CSC markers for different types of cancers have been identified, and with the appropriate technology, it is possible to focus on a subset of these specific markers that are highly expressed in these populations and in conjunction with other therapeutic strategies to target these specific CSC populations. A monoclonal antibody targeting CSC marker CD44 has been evaluated in two clinical trials (www.clinicaltrials.gov) in both solid and hematologic malignancies either alone or in combination with cytarabine, but the results of these studies are currently unknown [209, 210]. Similarly, several clinical trials were initiated recently that will test clinical utility of an antibody termed Tagraxofusp (SL-401) which targets CSC marker CD123 in patients diagnosed with hematologic malignancies [211].

**Table 1:** CSC markers from different types of cancers. Compilation of the most common CSC markers (in bold) identified from different types of cancers (references in brackets)

Tumor type	Markers
Leukemia	**CD34**^**+**^ [191, 196–198]; **CD38**^**−**^ [191, 197, 198]; **CD47**^**+**^ [191]; CCL-1 [191]; **CD96**^**+**^ [36, 191]; **CD90**^**−**^ [198]; **CD117**^**−**^ [198]; **CD133**^**+**^ [199]; **CD123**^**+**^ [191, 198]
Breast	**CD34**^**+**^ [200]; **CD24**^**low**^ [36, 48, 191, 197]; **ALDH1**^**+**^ [198]; **CD29**^**+**^ [201]; **Bmi-1**^**+**^ [202]; **CD133**^**+**^ [48]; **ESA**^**+**^ [191, 198]; **CD59**^**+**^ [36]
Pancreatic	**ESA**^**+**^ [48, 191]; **CD24**^**+**^ [48, 191, 198]; **CD44**^**+**^ [48, 191, 198]; **CD133**^**+**^ [36, 48, 198]
Lung	**CD44**^**+**^ [48, 191]; **CD133**^**+**^ [48, 191, 198, 203]; **CD59**^**+**^ [36]; **CD56**^**+**^ [203]
Liver	**ESA**^**+**^ [191]; **CD133**^**+**^ [48, 191, 198]; **CD90**^**+**^ [191, 198]; **CD44**^**+**^ [48, 191]; **CD176**^**+**^ [48]
Gastric	**CD133**^**+**^ [204]; **CD54**^**+**^ [48]; **CD44**^**+**^ [48, 191]
Colorectal	**ESA**^**+**^ [191]; **CD133**^**+**^ [36, 48, 191, 198, 205]; **CD166**^**+**^ [48, 191, 198]; **CD44**^**+**^ [48, 191, 198]; **CD24**^**+**^ [48, 191, 198]
Prostate	**Integrin α2β1** [48]; **CD44**^**+**^ [48, 198]; **CD133**^**+**^ [48, 198]; **Bmi-1** [206]
Melanoma	**CD34**^**+**^**/CD31**^**+**^ [200]; **CD20**^**+**^ [198]; **CD44**^**+**^ [207]
Ovarian	**CD44**^**+**^ [48, 197, 208]; **CD117**^**+**^ [48, 197, 208]; **CD133**^**+**^ [48, 208]; **CD24**^**+**^ [48]

### Targeting CSCs by blocking their stem cell niche and signaling pathways

The CSC microenvironment has been shown to be crucial for cancer initiation and tumor growth. It is not novel to try to target factors that potentiate tumor growth and dissemination as a therapeutic strategy; however, the problem is that these CSCs niches are enclosed and provide limited accessibility for most therapeutic antibodies or neutralizing cytokines. The use of exosomes in cancer therapy has gained more attention lately [212]. Exosomes are small intracellular membrane-based vesicles involved in the delivery by endocytosis or membrane fusion of molecules, antibodies, or drugs [213]. Exosomes expressing unique surface markers could be used to deliver drugs or molecules to a specific CSCs population to kill these cells [67]. Targeting specific factors that contribute to CSCs self-renewal and CSC proliferation by neutralizing those factors intracellularly or affecting different parts of the Hh, TGF-β, and Wnt pathway in several types of malignances is another approach to control CSCs [214]. Glasdegib is a small molecule which inhibits sonic hedgehog receptor smoothened and was recently approved by FDA in combination with low-dose cytarabine for newly diagnosed acute myeloid leukemia in patients with co-morbidities [215].

CSC microenvironment promote an immunosuppressive state and do not allow for a proper immune response, suggesting that one alternative approach to target this microenvironment is to blocking the action of myeloid suppressor cells action and targeting cellular receptors involved in immunomodulation, like PD1/PDL-1 [194]. For example, targeting VEGF-A with the humanized recombinant monoclonal antibody, bevacizumab. This drug inhibits the angiogenesis process by slowing down the growth of new blood vessels and disrupting the CSCs niche. It has already been shown in a glioblastoma murine model that treatment with bevacizumab depletes vasculature to the CSC microenvironment and drastically reduces the number of glioblastoma stem cells [216]. Fresolimumab, a monoclonal antibody that binds to all isoforms of TGFβ and thus modulates tumor microenvironment, has been tested in clinics in combination with focal irradiation for the treatment of metastatic breast cancers. This therapeutic combination was well tolerated and the subjects who received higher dose of the antibody had favorable antitumor responses and experienced longer median overall survival than the lower dose group [217].

Another way to block and reprogram CSCs is by using miRNAs. This has been shown by the use of exosomes to deliver specific miRNAs that block or arrest key CSCs signaling pathways [218]. SSCs express a unique set of miRNAs that maintain self-renewal, promote differentiation, and maturation. However, in CSCs, these miRNAs are deregulated. For example, the overexpression of miRNA-124 and miRNA-137 in human glioblastoma-derived cancer stem cells resulted in the loss of self-renewal and oncogenic capacity [219]. Han et al. also showed that blockage of miR-21 reverses EMT and CSC phenotype, suggesting that miRNA-21 has a role in CSC maintenance and this molecule could be targeted in future therapies [220]. No clinical trials utilizing such strategy are currently registered at clinicaltrials.gov but a number of promising pre-clinical studies have been published (reviewed in [221]).

## Concluding remarks

**Fig. 3:**
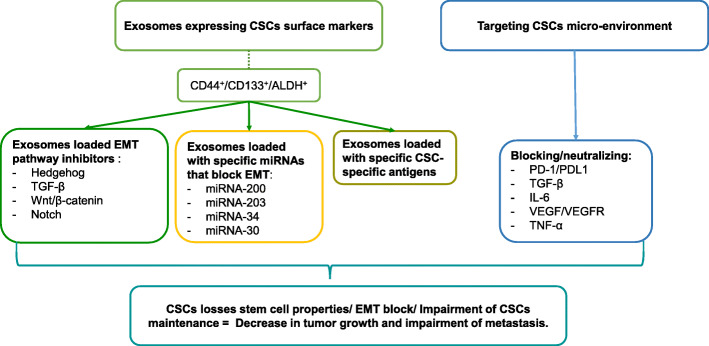
Potential therapeutic strategies to target CSCs. Specific CSC targeting could be achieved by creating exosomes that display CSC markers which can be used to deliver important CSC pathway inhibitors or to deliver miRNAs that block the EMT pathway. Alternatively, it is also possible to target CSCs by blocking their microenvironment preventing the formation of new CSCs

One of the major reasons for the failure of conventional cancer therapies is the presence of CSCs. Unfortunately, most drugs used in cancer therapy exert their effect by killing fast proliferating cancer cells, with the side effect of targeting fast proliferating normal cells. In addition to the severe side effects of traditional cancer therapies, drug therapy resistance and the failure in achieving long-term cancer remission are steering scientists into the research of developing targeted treatments. The discovery of a sub-population of tumor cells that have self-renewal and multipotent properties, now called cancer cell initiators or CSCs, opened new opportunities for research in the field of personalized cancer therapy. Understanding of the molecular and cellular mechanisms of CSCs is the best approach to efficiently target CSCs without affecting normal cells or SSCs (Fig. 3). The major pathways involved in CSCs are TGF-beta, Wnt, Hedgehog, and Notch, most of which are interconnected and participate not only in self-renewal properties but also in EMT initiation, an important factor in cancer initiation and metastasis. Therefore, targeting multiple mechanisms involved in growth and maintenance of CSCs is definitively the best path toward a safe and more effective cancer therapy.

## Data Availability

The data materials supporting the current study are included within the article.

## Abbreviations

CSCs: Cancer stem cells
SSCs: Somatic stem cells
ESCs: Embryonic stem cells
MSCs: Mesenchymal stromal cells
AML: Acute myelogenous leukemia
CD: Cluster of differentiation
ALDH1: Aldehyde dehydrogenase 1
ESA: Epithelial-specific antigen
NOD/SCID: Non-obese diabetic severe combined immunodeficiency
PAMPs: Pathogen-associated-molecular patterns
NF-κB: Nuclear factor kappa B
TLRs: Toll-like receptors
ZEB1: Zinc finger E-box-binding homeobox1
EMT: Epithelial-mesenchymal transition
MET: Mesenchymal-to-epithelial transition
Treg: Regulatory T cell
GVHD: Graft-vs-host disease
ARDS: Acute respiratory distress syndrome
HLAs: Human leukocyte antigens
TAM: Tumor-associated macrophages
STATs: Signal transducers and activators of transcription
IRF-5: Interferon regulatory factor-5
NK: Natural killer
MHC: Major histocompatibility complex
JAKs: Janus family tyrosine kinases
DLLs: Delta-like ligands
Hh: Sonic hedgehog or hedgehog
TWIST1: Twist-related protein 1
miRNAs: MicroRNAs
ALDH: Aldehyde dehydrogenase
